# Comparison of RNA localization during oogenesis within *Acipenser ruthenus* and *Xenopus laevis*

**DOI:** 10.3389/fcell.2022.982732

**Published:** 2022-09-20

**Authors:** Viktoriia Iegorova, Ravindra Naraine, Martin Psenicka, Monika Zelazowska, Radek Sindelka

**Affiliations:** ^1^ Laboratory of Gene Expression, Institute of Biotechnology of the Czech Academy of Sciences, Vestec, Czechia; ^2^ Faculty of Fisheries and Protection of Waters, South Bohemian Research Center of Aquaculture and Biodiversity of Hydrocenoses, University of South Bohemia in Ceske Budejovice, Vodnany, Czechia; ^3^ Department of Developmental Biology and Morphology of Invertebrates, Institute of Zoology and Biomedical Research, Faculty of Biology, Jagiellonian University, Krakow, Poland

**Keywords:** oogenesis, RNA localization, *Xenopus laevis*, *Acipenser ruthenus*, TOMO-Seq

## Abstract

The oocyte is a unique cell, from which develops a complex organism comprising of germ layers, tissues and organs. In some vertebrate species it is known that the asymmetrical localization of biomolecules within the oocyte is what drives the spatial differentiation of the daughter cells required for embryogenesis. This asymmetry is first established to produce an animal-vegetal (A-V) axis which reflects the future specification of the ectoderm, mesoderm, and endoderm layers. Several pathways for localization of vegetal maternal transcripts have already been described using a few animal models. However, there is limited information about transcripts that are localized to the animal pole, even though there is accumulating evidence indicating its active establishment. Here, we performed comparative TOMO-Seq analysis on two holoblastic cleavage models: *Xenopus laevis* and *Acipenser ruthenus* oocytes during oogenesis. We found that there were many transcripts that have a temporal preference for the establishment of localization. In both models, we observed vegetal transcript gradients that were established during either the early or late oogenesis stages and transcripts that started their localization during the early stages but became more pronounced during the later stages. We found that some animal gradients were already established during the early stages, however the majority were formed during the later stages of oogenesis. Some of these temporally localized transcripts were conserved between the models, while others were species specific. Additionally, temporal *de novo* transcription and also degradation of transcripts within the oocyte were observed, pointing to an active remodeling of the maternal RNA pool.

## 1 Introduction

The oocyte is a unique cell, whereby following its fertilization, the resulting embryo develops to produce hundreds of cell types that are later organized into many tissues and organs (reviewed by Houston, 2013). The oocyte of many species already contains all the necessary materials (e.g., the yolk and mitochondria) needed to maintain its metabolism, and also to control development during the early stages of embryogenesis. Transcripts and proteins which are accumulated during oogenesis are used by the developing embryo until zygotic genome activation (ZGA) and regulate the early stages of embryogenesis (reviewed by Gilbert, 2000; reviewed by Kim and Lee, 2014; Peshkin et al., 2015).

The process of oocyte formation starts from the primordial germ cells (PGCs) development in the gonadal ridge, arrest of meiocytes at meiosis I, oocyte growth, continuation with ovulation and the resumption of meiosis, fertilization and then the beginning of the new life cycle (reviewed by Jagarlamudi and Rajkovic, 2012). In comparison to somatic cells, the oocyte is much larger, which makes studying the events of RNA localization easier (King et al., 1999). The mechanism of oogenesis varies among different species. In some organisms, like sea urchins, frogs, and fishes (eg. *Danio rerio*), the females produce hundreds to thousands of oocytes in a short period of time. In contrast, other species including humans and most mammals, produce limited number of oocytes during their lifetime (reviewed by Gilbert, 2000; reviewed by Spence et al., 2008). The number of stages that defines the oogenesis can vary between certain species. For example, based on morphological criteria and on physiological and biochemical events, 14 stages can be distinguished in fruit fly (*Drosophila melanogaster*), from the start of the formation of the egg chamber/follicle, which consists of a cyst of germ cells surrounded by somatic follicle cells, to the final matured egg (reviewed by Xu and Gridley, 2012). Five stages can be found in *D. rerio,* starting from the pre-follicle phase of primary growth followed by the cortical alveolus stage, vitellogenesis, oocyte maturation and finally the matured egg (Selman et al., 1993).

One of the most common models used for the study of oogenesis are the *Xenopus* species. *Xenopus* oogenesis is constant in the adult ovary, with continuously differentiating oogonia into oocytes. Oogenesis is not synchronized and therefore females contain oocytes of different stages. A cycle of oogenesis is considered completed when a large number of oocytes are at the stage VI (reviewed by Rasar and Hammes, 2006). *Xenopus* has continued to be an attractive model because it is easy to obtain, maintain, and produces large oocytes that can be easily manipulated and orientated due to the presence of pigmented granules at the animal pole. Currently there are nearly a thousand published studies about RNA localization using *X. laevis* oocytes (reviewed by Dettlaff and Rudneva, 1991; reviewed by Rasar and Hammes, 2006; National Library of Medicine, 2002).

Raikova (1973), Raikova (1974) pointed out that during oogenesis, the frog oocytes share strong cytological similarities with sturgeons including sterlets (*Acipenser ruthenus*), such as the nucleolar and other nuclear structures, cytoplasmic organelles, the same structure of yolk platelets, presence of cortical granules, absence of ribosomes in previtellogenesis, extrusions of nucleolar material into the cytoplasm, and the same dense material cementing the mitochondria. The oogenesis of *X. laevis* and sturgeon comprises of six stages and can be categorized into three phases of oocyte development: pre-vitellogenesis, vitellogenesis (accumulation of yolk) and post-vitellogenesis/choriogenesis (deposition of egg envelopes) (Dumont, 1972; Dettlaff et al., 2006; Zelazowska, 2010; reviewed by Zelazowska and Fopp-Bayat, 2017; Zelazowska and Fopp-Bayat, 2019). *Xenopus* oocytes at the pre-vitellogenic phase (stage I) are transparent with a size between 50–300 µm. Vitellogenic phase takes place from stages II to V with sizes: 300–450 µm for stage II, 450–600 µm for stage III, 600–1000 µm for stage IV, and 1000–1200 µm for stage V. The visible animal - vegetal (A-V) axis is distinguishable at the stage III and is due to the pigment granules accumulated on the animal pole. Post-vitellogenic *X. laevis* eggs have sizes between 1200–1300 µm (Dumont, 1972). Although sturgeon oogenesis also passes through six developmental stages, their morphological descriptions are weak. The size of the matured sterlet egg ranges between 1900–2500 µm (Dettlaff et al., 2006), but the sizes of the developing sterlet oocytes have not yet been described. Oocyte polarization index (PI) is used in the sturgeon hatcheries to estimate the stage of sexual maturity of females. The PI is calculated by finding the ratio between the distance separating the animal pole and the germinal vesicle versus the distance between the animal to the vegetal pole (Chebanov and Galich, 2013).

During early embryonic development, sterlets, as well as *Amphibians*, represent a completely dividing (holoblastic) embryo (Dettlaff et al., 2006; reviewed by Elinson and del Pino, 2012). The development of the germ layers of many organisms is specified and predetermined by maternal RNAs and proteins that are localized unevenly along the A-V axis during oogenesis (reviewed by Marlow, 2010; reviewed by Owens et al., 2017)*.* In *X. laevis*, the first sign of asymmetry is visible at stage I of oogenesis, whereby a spherical structure is localized in close proximity to the nucleus of the oocyte. This structure, referred to as the Balbiani body or mitochondrial cloud (MC), contains mitochondria, Golgi complex, endoplasmic reticulum, lipids and pigment granules (Heasman et al., 1984; reviewed by Kloc et al., 2004). Two localization pathways that contribute to vegetal localization have been described for the *X. laevis* oocytes: the METRO (MEssage TRansport Organizer) and the Late pathway (Kloc and Etkin, 1995) (Table 1). Transcripts that are localized in the MC utilize the METRO pathway and migrate towards the future vegetal pole to define the A-V polarity of the oocyte from the first stages of oogenesis. By the stage II-IV, the METRO pathway localizes transcripts, including *nanos1* (previously *nos1*/*xcat2*), *dazl* (previously *Xdazl*)*, pgat* (previously *Xpat*), *Xlsirts, ddx25* (previously *xcat3*), to the vegetal pole (Kloc et al., 1993; Forristall, et al., 1995; Kloc and Etkin, 1995, 1998; reviewed by King et al., 1999). Transcripts, utilizing the late pathway (eg. *gdf1* (previously *vg1*)*, vegt*) are distributed throughout the cytoplasm at stage I, and do not use the MC. By stage III-VI these transcripts start to migrate towards the vegetal pole and anchor at a broader region of the vegetal cortex (Forristall et al., 1995; Kloc and Etkin, 1995, 1998; reviewed by King et al., 1999; Houston, 2013). An equivalent of the Balbiani body, the Balbiani cytoplasm was also described in the sturgeon species. Granular (Balbiani) ooplasm of *A. gueldenstaedtii* oocytes and *X. laevis* Balbiani bodies share similarities presented in their molecular composition and ultrastructure (Zelazowska et al., 2007). The Balbiani cytoplasm contains nuage aggregations, mitochondria and is detected around the germinal vesicle in stage I. During oocyte development, the Balbiani cytoplasm expands toward the oocyte periphery and nuage aggregations become uniformly dispersed throughout the entire ooplasm (Zelazowska et al., 2007).

**TABLE 1:** Localization of maternal transcripts in the oocytes of the *Xenopus laevis*.

Pathway	RNAs	Stages and localization	References
Early pathway/METRO	*Xdazl*	I-uniform, II-IV-migration to the vegetal pole	1
	*dazl*	I-MC, III-IV-vegetal pole	2
	*Xcat2*	I-MC, but still in the cytoplasm, IV-vegetal pole	1
	*Xcat2*	I-MC, II-vegetal pole	3
	*Xcat2*	I-MC, but still in the cytoplasm, II-MC, vegetal pole	4
	*Xlsirt*	I-MC and cytoplasm, II-vegetal pole	4
	*Xlsirt*	II-MC, III-vegetal pole	5
	*Xlsirt*	II-MC, IV-vegetal pole	6
Late pathway	*Vg1*	II-III-around the nucleus, III-VI-migration to the vegetal pole	1
	*Vg1*	I-II-everywhere, III-IV-vegetal pole	3
	*Vg1*	I-cytoplasm, II-migration to the vegetal pole, III-vegetal pole with continuing streaming	4
	*Vg1*	I-everywhere, III-vegetal pole (but still migrating)	5
	*Vegt*	I-uniformly distributed, IV-vegetal pole	2; 7
Intermediate pathway	*fatvg*	I-cytoplasm, II-MC, III-V-migration to the vegetal pole	8
	*Hermes*	I-cytoplasm, early II-MC, II-III-accumulation at the vegetal cortex, IV-vegetal cortex	9
Animal pathway	*PABP*	VI	10
	*β-tubulin*	VI	11
	*An1*	I-III-uniform, IV-animal pole	12
	*An2*	I-III-uniform, IV-animal pole	12
	*An3*	I-III-uniform, IV-animal pole	12
	*An4a*	VI	12
Summary scheme of the oocyte stages where the majority (darker shades) of the transcript relocalization is occurring			
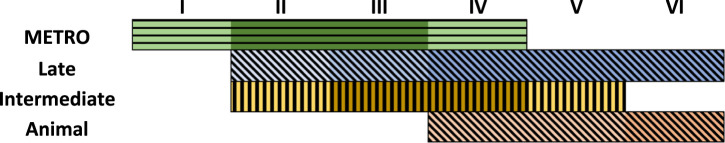			

**References:** 1. Reviewed by King et al. (1999), 2. Houston (2013), 3. Forristall et al. (1995), 4. Kloc and Etkin (1995), 5. Kloc and Etkin (1998), 6. Kloc et al. (1993), 7. Zhang and King (1996), 8. Chan et al. (1999), 9. Zearfoss et al. (2004), 10. Schroeder and Yost (1996), 11. Yisraeli et al. (1990), 12. Reviewed by Schnapp et al. (1997).

Some studies have also described an “intermediate” pathway in *X. laevis*, which is a combination or overlap of the METRO and Late pathway. Transcripts of this pathway (eg. *plin2* (previously *fatvg*)*, dnd1*, *grip2*, *trim36*, *rbpms/rbpms2* (*previously hermes*)) enter the Balbiani body in stage II oocytes and distribute at the wide area of the vegetal cortex of the oocytes during stages IV-V (Chan et al., 1999; Zearfoss et al., 2004; reviewed by Houston, 2013).

Compared to the well-studied vegetal pole, very little is known about the transcripts that are localized to the animal pole. A few studies have already demonstrated that during early oocyte development, transcripts which belong to the animal pathway are uniformly distributed. The animal localization can be first observed at stage IV in *X. laevis*. Transcripts coding *pabpc4* (previously *PABP*)*, tubb* (previously *β-tubulin*) and *nup93* (previously *An4a*) were detected in the animal pole at VI stage only (Yisraeli et al., 1990; Schroeder and Yost, 1996; reviewed by Schnapp et al., 1997). Contrary to the few animal transcripts that have been described in the past, many recent studies have demonstrated that there are in fact hundreds of animally localized transcripts (Claußen et al., 2015; Owens et al., 2017; Sindelka et al., 2018; Naraine et al., 2022). Additionally, our recent study has shown that many of these maternal animal transcripts show a high level of conserved localization amongst diverse species (*D. rerio, A. ruthenus, X. laevis* and *Ambystoma mexicanum*) (Naraine et al., 2022). We selected the two best models (easily accessible and early oocyte coloring reflecting A-V axis) to study RNA localization during oogenesis at the complete transcriptome level. In this research, we utilized spatial RNA sequencing (TOMO-Seq) to study oocytes from early and advance vitellogenic stages in *Actinopterygii* (*A. ruthenus*) and III-V oocyte stages in *Amphibia* (*X. laevis*).

## 2 Materials and methods

### 2.1 Ethics approval

All experimental procedures involving model organisms were carried out in accordance with the Czech Law 246/1992 on animal welfare. *Acipenser ruthenus* females were kept in the Research Institute of Fish Culture and Hydrobiology in Vodnany, Czech Republic and protocols were reviewed by the Animal Research Committee of the Faculty of Fisheries and Protection of Waters, South Bohemian Research Center of Aquaculture and Biodiversity of Hydrocenoses, Research Institute of Fish Culture and Hydrobiology, Vodnany, Czech Republic. The *X. laevis* females were from the colony of the Institute of Biotechnology and protocols were approved by the animal committee of the Czech Academy of Sciences.

### 2.2 Sample collection

*Xenopus laevis* females were anesthetized with benzocaine for 1 h. Their abdomen was then opened with surgical scissors and the oocytes were placed into High Salt Solution (HSS). Oocytes were observed under the microscope, manually separated, and visually divided by size from the smallest oocytes with signs of pigmentation on the animal pole (stage III) to the late stage V (big oocytes). Collected oocytes had sizes from 560 to 1200 µm. Four groups were created: very small oocytes (stage III, size 560–580 µm); small oocytes (stage IV, size 720–810 µm), medium (early stage V, size 1050 µm) and big (late stage V, size 1140–1200 µm).

Different stages of *A. ruthenus* oocytes were collected by biopsy of matured females (5–8 years old). The samples contained small, individual ovarian follicles in which early previtellogenic, diplotene stage oocytes (according to Zelazowska and Fopp-Bayat, 2019) grew, as well as large ovarian follicles. The nucleoplasm of all previtellogenic oocytes contained lampbrush chromosomes and multiple nucleoli. In the vicinity of the nucleus the granular ooplasm and lipid body were present. In the large follicles the vitellogenic, pigmented oocytes developed. Two groups of vitellogenic *A. ruthenus* oocytes were created: small (early vitellogenic, size 1320–1500 µm) and big (advanced vitellogenic, size 1920–2100 µm) (Supplementary Figure S1).

Light and electron microscopy were used to characterize the stages of interest for the *A. ruthenus* oocytes. Samples of *A. ruthenus* ovaries were fixed for 2 weeks in 2.5% glutaraldehyde (POCH) in 0.1 M phosphate buffer (pH 7.3). Then, they were rinsed and postfixed in 1% osmium tetroxide in 0.1 M phosphate buffer (pH 7.3) containing saccharose (5.6 g in 100 ml). Next, they were dehydrated in a series of ethanol and acetone and embedded in glycid ether 100 (Serva Electrophoresis). Semi-thin sections (0.7 µm) were stained with methylene blue in 1% borax and photographed using a Leica DMR light microscope. Ultrathin sections (90 nm) were contrasted with uranyl acetate and observed in a transmission electron microscope (JEOL JEM 2100 in the Laboratory of Microscopy, Department of Cell Biology and Imaging, Institute of Zoology and Biomedical Research, Jagiellonian University) at 80 kV. The ovarian follicles were also photographed under a Nikon SMZ 1500 (Tokyo, Japan).

To detect nucleus position in the early and advanced vitellogenic eggs, sampled oocytes were fixed at least for 24 h in a freshly made medium (10 ml 99% acetic acid with 30 ml 32–35% formaldehyde with 60 ml 70–98% ethanol per 100 ml of Serra solution). The fixed oocytes were rinsed with tap water and were sectioned into two parts with a razor blade in the plane of the longitudinal axis (Rodina, 2006).

Early and advance vitellogenic stages of *A. ruthenus* and III-V oocyte stages of *X. laevis* were embedded in Tissue-Tek O.C.T. Compound, oriented using delicate forceps along the A-V axis (animal pole positioned at the top) and immediately frozen on dry ice and stored at -80°C.

### 2.3 Sample preparation

Samples were incubated for 10 min in the cryostat chamber (−20°C) and then cut into 30 μm slices along the A-V axis. Number of obtained slices were counted, equally distributed in sequential order into five tubes with the same number of slices per tube. The diameter of the oocyte was calculated by multiplying the thickness of the slices by the number of slices obtained. Tubes were then labelled to correspond to the relevant segments of the oocyte (A—extremely animal, B—animal, C—central, D—vegetal, E—extremely vegetal).

Total RNA was extracted using Qiagen RNeasy Minikit according to the manufacturer’s instructions. The concentration of total RNA was measured using a spectrophotometer (Nanodrop 2000; ThermoFisher Scientific), and the quality of RNA was assessed using a Fragment Analyzer (AATI, Standard Sensitivity RNA analysis kit, DNF-471). No signs of RNA degradation were observed. Absence of inhibitors and the precision of the orientation of the embedded oocyte were evaluated using RT-qPCR quantification of the RNA spike (TATAA Biocenter) and several known localized marker (animal and vegetal) transcripts respectively.

The cDNA was prepared using total RNA (*X. laevis:* 20–50 ng, *A. ruthenus:* 20–50 ng), 0.5 μl of oligo dT and random hexamers (50 μM each), 0.5 μl of dNTPs (10 mM each) and 0.5 μl of RNA spike (TATAA Universal RNA Spike, TATAA Biocenter), which were mixed with RNAse free water to a final volume 6.5 μl. Samples were incubated for 5 min at 75°C, followed by 20 s at 25°C and cooling to 4°C. In the second step, 0.5 μl of SuperScript III Reverse Transcriptase (Invitrogen), 0.5 μl of recombinant ribonuclease inhibitor (RNaseOUT, Invitrogen), 0.5 μl of 0.1 M DTT (Invitrogen), and 2 μl of 5 × First strand synthesis buffer (Invitrogen) were added and incubated: 5 min at 25°C, 60 min at 50°C, 15 min at 55°C and 15 min at 75°C. Obtained cDNAs were diluted to a final volume of 100 μl and stored at −20°C.

### 2.4 Primer design and quantitative PCR

Primer assays of selected maternal transcripts were designed using NCBI Primer-Blast (https://www.ncbi.nlm.nih.gov/tools/primer-blast/) (Ye et al., 2012). Expected amplicon length was set to 97–187 bp and Tm to 60°C. Primer sequences are available in Supplemental Material S2: Supplementary Table S1. The RT-qPCR reaction contained 3.5 μl of TATAA SYBR Grand Master Mix, 0.29 μl of forward and reverse primers mix (mixture 1:1, 10 μl each), 2 μl of cDNA and 1.21 μl of RNase-free water in 7 μl final volume. qPCR was performed using the CFX384 Real-Time system (BioRad) with conditions: initial denaturation at 95°C for 3 min, 45 repeats of denaturation at 95°C for 15 s, annealing at 60°C for 20 s and elongation at 72°C for 20 s. Melting curve analysis was performed after to evaluate reaction specificity and only one product was detected for all assays. Only samples with continuous gradient profiles of the marker transcripts were selected for library preparation.

### 2.5 Library preparation

Libraries were prepared using 100 ng (big *A. ruthenus* oocytes, *n* = 5), 25 ng (small *A. ruthenus* oocytes *n* = 5) and 50 ng (all *X. laevis* oocytes; big *n* = 2 (stage late V), medium *n* = 2 (stage early V), small *n* = 5 (stage IV), very small *n* = 3 (stage III)) of total RNA. Samples were depleted using RiboCop rRNA Depletion kit (Lexogen) and libraries were prepared using SureSelect XT (Agilent). Optimum PCR cycles (13–17) were determined based on starting RNA amount during library preparation. Library qualities were assessed using the Fragment Analyzer (AATI, NGS High Sensitivity kit (DNF-474) and the concentrations were determined by the Qubit 4 Fluorometer (ThermoFisher Scientific). Equimolar library pools were prepared and sequenced using two 2x80 bp, NextSeq 500 runs. Obtained sequencing yield were on average 6.8 M reads in *A. ruthenus* and 5.5 M reads in *X. laevis*.

### 2.6 Preprocessing of RNA-Seq data

RNA-Seq reads were processed as previously described in Naraine et al. (2022). In brief, TrimmomaticPE (v. 0.36) (Bolger et al., 2014) was used to remove adaptor sequences and low-quality reads, while SortMeRNA (v. 2.1b) (Kopylova et al., 2012) was used to remove mtRNA reads (GenBank id: NC_001573.1, KF153104.1) and any remaining rRNA reads. The *X. laevis* reads were then aligned to the *X. laevis* v9.1 genome (Fortriede et al., 2020) using STAR v2.5.2b (Dobin et al., 2013) and counted using htseq-count. The *A. ruthenus* reads were pseudoaligned using kallisto (v. 0.43.1) (Bray et al., 2016), to the initial *A. ruthenus de novo* transcriptome from Naraine et al. (2020). The data were deposited in the National Center for Biotechnology Information’s Gene Expression Omnibus (GEO).

### 2.7 RNA-Seq analysis

Transcripts were initially filtered to remove those that had zero counts across all samples. The DESeq2 (v. 1.32.0) package was used to normalize the transcript counts (median-of-ratios method) and detect differentially localized transcripts (DLTs) between the sections or oocyte stages using the Likelihood Ratio Test (LRT) (Love et al., 2014). To identify transcript alterations between the sections or stages the following design models were used:

1) Alteration between the sections at the same stage:
  a) design: ∼replicate + position; reduced design: ∼replicate.
2) Alteration in the profiles across the different stages:
  a) Transcripts with altered profiles: design: ∼Size + position + Size:position; reduced: ∼Size + position
  b) Transcripts with altered magnitudes: design: ∼Size + position; reduced: ∼Size
3) Alteration of the total transcript count between the different stages
  a) design: ∼Size; reduced: ∼1; uses the sum of the normalized counts for each sample as input counts

Principal component analysis (PCA) was used to visually inspect the replicates and identify if there were any observable differences between the stages and the sections. DLTs were defined as those with an adjusted *p*-value (padj) value less than 0.1 and also a total transcript count greater than 20 within the whole oocyte. The transcript counts for each section was summarized as a percentage relative to the total transcripts within the oocyte. These relative expression values were used to categorize the DLTs into localization profiles based on defined thresholds as previously defined (Sindelka et al., 2018; Naraine et al., 2022). The profiles comprised of the extreme animal, animal, central, vegetal, and extreme vegetal profiles. Categorization of any alterations in the profiles of the DLTs across the different stages was done using the degPatterns function from DEGreport (v. 1.28.0) package (Pantano, 2021). The “group difference” option was used to select for DLTs that showed at least a 1.5x or 2x fold change difference between either the stages or the sections, while the “summarize” option was used to identify those that were also reproducible across replicates. The profiles were verified manually and some clusters re-analyzed using optCluster (v. 1.3.0) with the “Diana” clustering algorithm (Sekula et al., 2017). The transcripts were annotated using the annotations derived from the previous ortholog analysis done by Naraine et al. (2022). In brief, protein sequence similarity and gene symbol matching were used to find the most probable orthologs between the two models. An orthologous unit number was used to denote orthologous genes in the absence of a common gene symbol.

Gene Ontology terms associated with the genes were derived using the gprofiler2 package (v. 0.2.1) (Raudvere et al., 2019) with the default parameters except for correction method = “bonferroni”, user threshold = “0.05”, domain scope = “annotated”, background organism = “*H. sapiens”* and significance = “FALSE”.

## 3 Results

**FIGURE 1:**
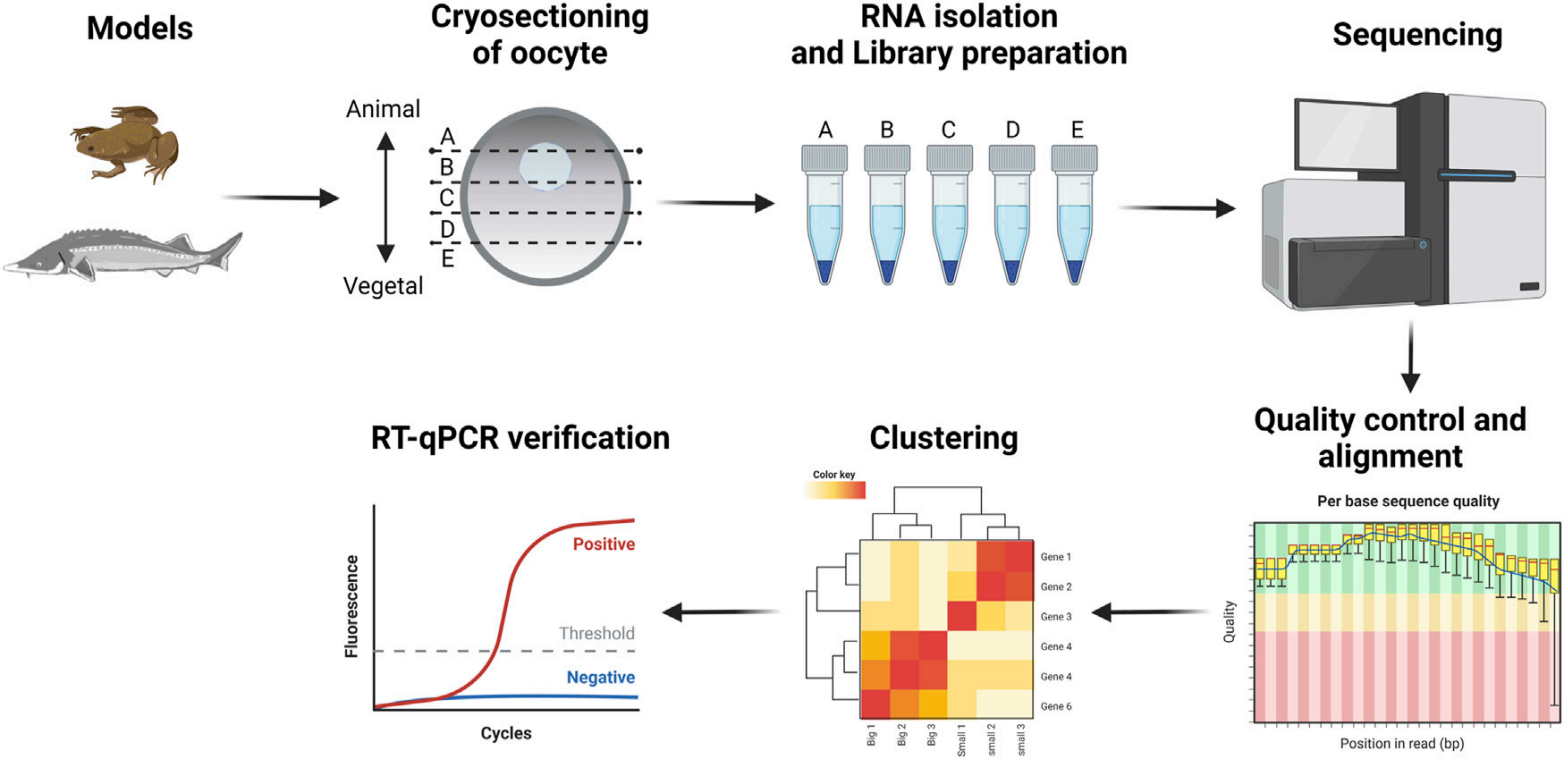
Schematic of the methodological workflow.

**FIGURE 2:**
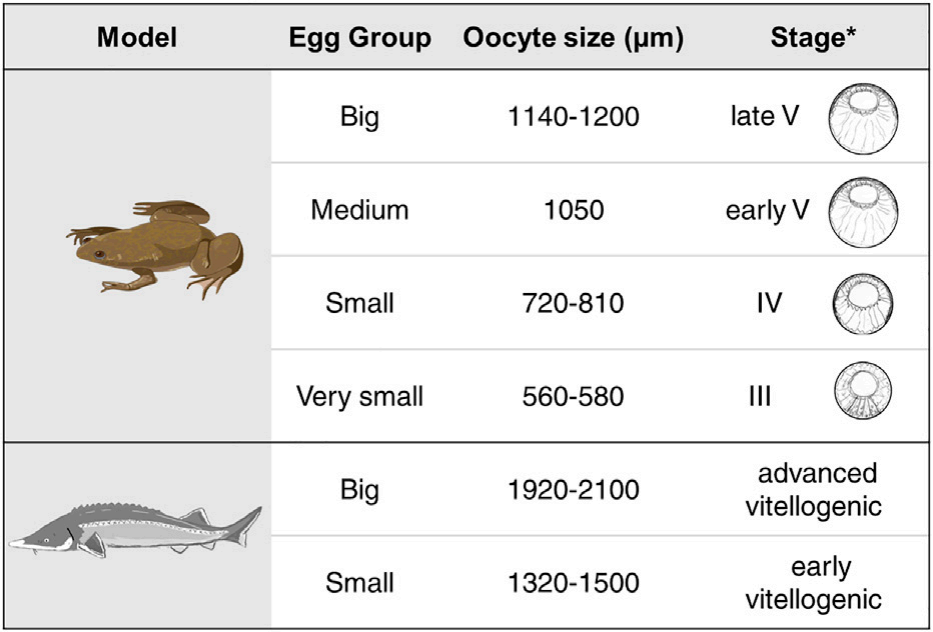
Summary of the different oocyte sizes assessed for each of the models. Shown for the *X. laevis* model are the schematic drawings for the equivalent stages for the given oocyte as derived from *Carotenuto and Tussellino (2018). Stage classification of the *X. laevis* oocytes are from Dumont (1972).

We studied RNA localization during oogenesis using the TOMO-Seq approach in five (A - extremely animal, B - animal, C - central, D - vegetal, E - extremely vegetal) sections along the A-V axis (Figure 1). Oocytes of *X. laevis* were divided into four categories reflecting already known stages (III stage - very small, IV stage - small, early V stage–medium and late V - big) while *A. ruthenus* oocytes were divided into two categories (early vitellogenic-small and advanced vitellogenic-big) (Figure 2).

Microscopic analysis was used to characterize the differences between the early and advanced vitellogenic oocytes of the *A. ruthenus*. There was a distinct size difference between these two stages (Supplementary Figure S1). Microscopic observations showed in the early previtellogenic oocyte that the nuclear region was much larger than in the mid‐previtellogenic oocyte. This large nuclear region in the early pre-vitellogenic oocyte encompassed a large area within the central region of the oocyte while in the mid-previtellogenic oocyte the nuclear region was more condensed and animally localized, with large spaces flanking both sides (Supplementary Figure S2). In the small (early vitellogenic) oocytes the nucleus was located in the center while in the big (advanced vitellogenic) oocytes, the nucleus was in the animal hemisphere (Supplementary Figure S1).

**FIGURE 3:**
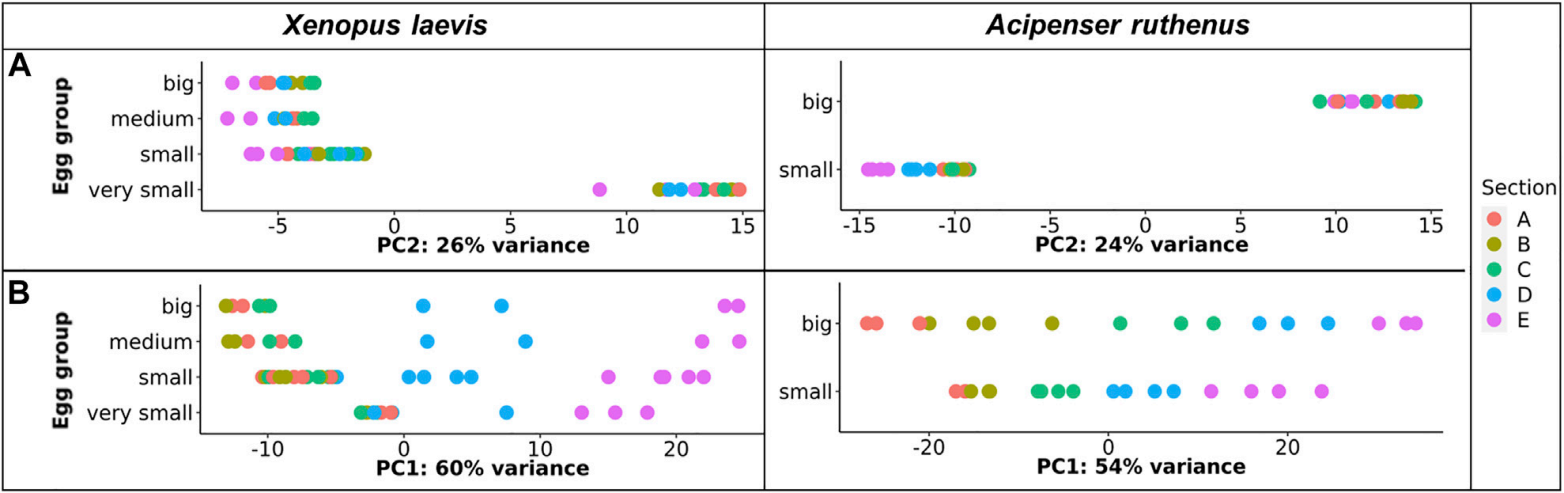
PCA plot showing the top 500 most variable genes for each of the different oocyte sizes from the models. **(A)** Principle component 2 showing variation in transcript as a function of oocyte size. **(B)** Principle component 1 showing variation in transcript across segment and size. The sections correspond to the regions of the oocyte that were cryosectioned, whereby sections A - extremely animal, B - animal, C - central, D - vegetal, E - extremely vegetal.

TOMO-Seq results were analyzed by in-house workflow and the PCA of the 500 most variable transcripts revealed that the greatest difference was between the very small oocytes and the larger (small, medium, big) oocytes of the *X. laevis* (Figure 3A). A similar difference was found between *A. ruthenus* small and big oocytes (Figure 3A). Analysis of the PC1 reflected variation among the individual sections and showed that the largest variations were observable relative to the size of oocyte suggesting a continuous increase of RNA asymmetry during oogenesis (Figure 3B).

More than 15000 or 21000 different transcripts (>20 counts in oocyte) were identified in *X. laevis* or *A. ruthenus* oocytes, respectively (Table 2). DLTs were identified as described in the methods, followed by categorization of the transcript profiles. On average more than 1000 DLTs were identified per oocyte stage, with the exception of 450 DLTs in the very small *X. laevis* oocyte. The numbers of DLTs were larger in the *A. ruthenus*, suggesting a more polarized oocyte (Table 2). Relatively similar numbers of DLTs were found in the animal and vegetal localization groups (Table 2). We compared the number of identified DLTs and their localization profiles observed in the big oocytes relative to the matured *X. laevis* and *A. ruthenus* eggs published by Naraine et al. (2022). There was a high concordance (>96%) between the identified animal (animal and extreme animal) and ∼50% of vegetal transcripts with the previous results for both *X. laevis* and *A. ruthenus*.

**TABLE 2:** Number of differentially localized transcripts that are overabundant in the five defined localized profiles.

	*Xenopus laevis*				*Acipenser ruthenus*	
	Big	Medium	Small	Very small	Big	Small
Total transcripts	16,045	16,076	15,832	15,952	22,591	21,862
DLTs	1,331	1,248	1,714	450	13,901	3,184
Annotated DLTs	1,127	1,068	1,482	359	7,550	1,847
Extreme animal	381	424	506	53	3,456	908
Animal	20	12	57	10	1,684	15
Center	0	0	1	2	5	31
Vegetal	444	502	229	98	4,280	639
Extreme vegetal	205	164	142	91	363	263
Undefined	281	146	779	196	4,113	1,328

DLTs are defined by <0.1 padj and >20 transcripts/oocyte.

### 3.1 Multiple profile changes were detected in both species

Transcript changes across the oocyte stages were assessed as either a change in the profile across the stages or a change in the magnitude of the transcript within a given segment across the stages. The identified DLTs were then further filtered during clustering to identify those with greater than a 1.5x fold change difference between any given segment between the stages. In *X. laevis*, 1479 DLTs (Supplementary Material S2: Supplementary Table S2) were observed that had sub-sectional alterations across the stages, while in *A. ruthenus* 10225 (Supplementary Material S2: Supplementary Table S3). Given the large quantity of DLTs in *A. ruthenus*, a fold change of 2x was used instead, and resulted in the detection of 4743 DLTs instead. The cluster profiles of these filtered DLTs were then assessed to group together transcripts that had either already established their localization profile during the early stages of oogenesis or instead during the late stages.

#### 3.1.1 Vegetally localized transcripts

**FIGURE 4:**
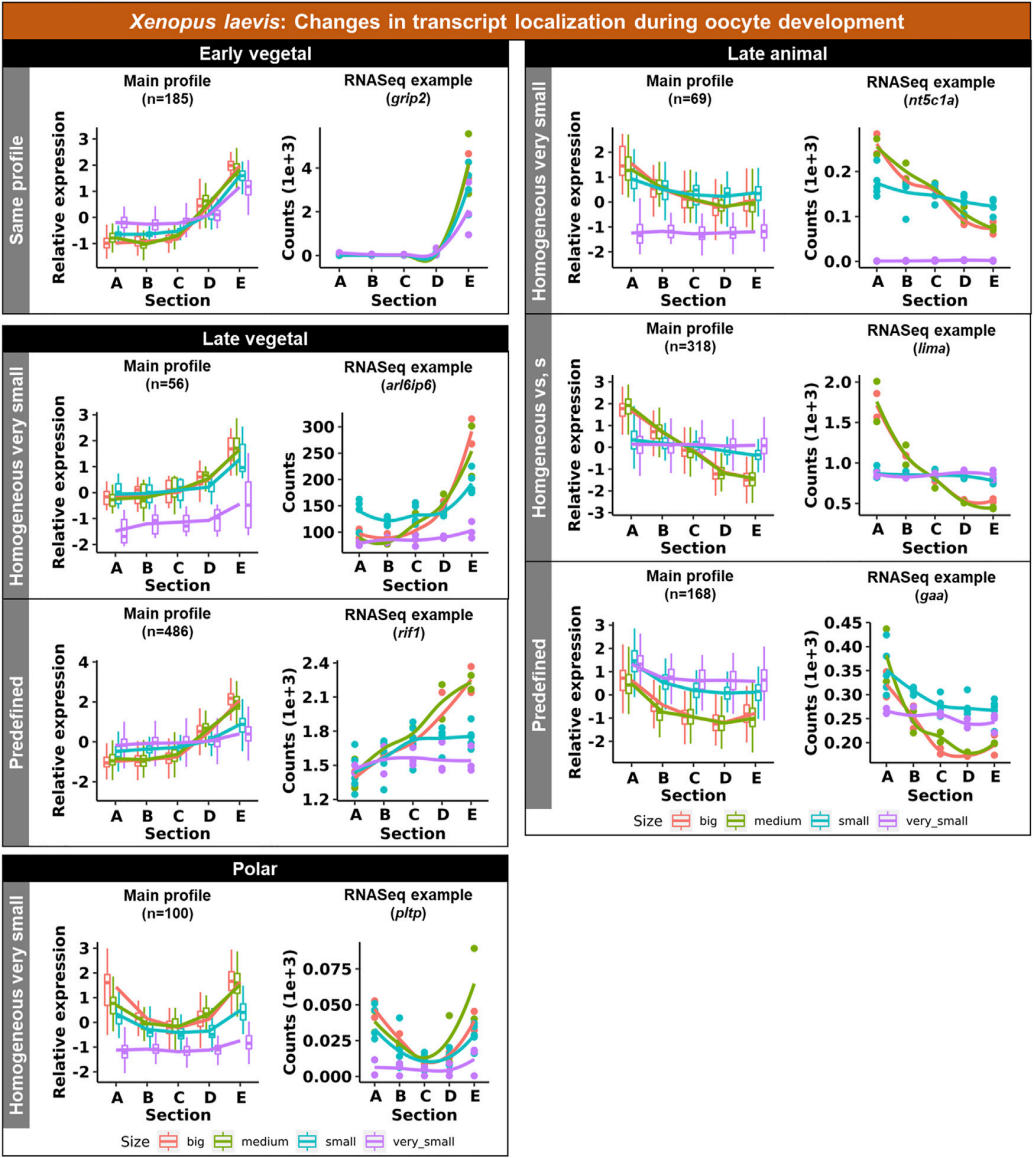
Unique groups representing the significant transcript sub-cellular alterations between the different oocyte sizes of the *Xenopus laevis.* Four groups of localization profiles were observable, early vegetal, late vegetal, late animal and polar. The following subgroups were observed: same profile - similar profile in early and late stages; predefined - profile already established in the early stages; homogeneous very small/small - profile is ubiquitous in the small/very small stage; homogeneous vs., s - profile is ubiquitous in both the small and very small stage. The sections correspond to the regions of the oocyte that were cryosectioned, whereby sections A - extremely animal, B - animal, C - central, D - vegetal, E - extremely vegetal.

**FIGURE 5:**
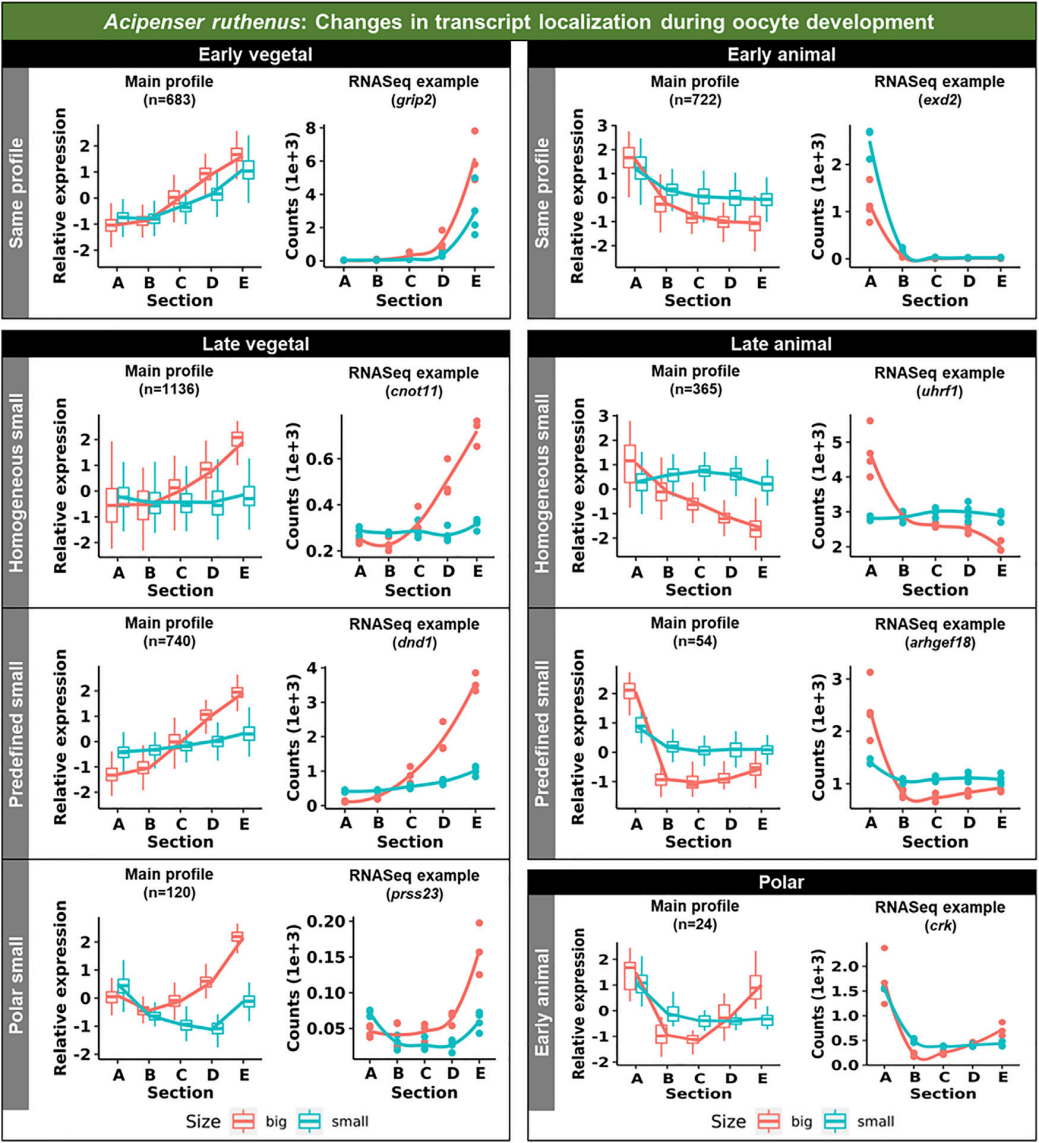
Unique groups representing the significant sub-cellular transcript alterations between the different oocyte sizes of the *Acipenser ruthenus*. Five groups of localization profiles were observable, early vegetal, late vegetal, early animal, late animal and polar. The following subgroups were observed: same profile - similar profile in early and late stages; predefined small - profile already established in the early stage; homogeneous small - profile is ubiquitous in the small stage; polar small—profile shows maximum expression in the polar regions of the oocyte; early animal—profile shows animal distribution in the early stage. The sections correspond to the regions of the oocyte that were cryosectioned, whereby sections A - extremely animal, B - animal, C - central, D - vegetal, E - extremely vegetal.

Vegetal transcripts are key for early development and the functions of many of them have already been elucidated during the last decades. Several changes in the vegetal profiles can be observed in both models. The localization of the vegetal transcripts can be described as either “early vegetal” or “late vegetal”. In the early vegetal group, the profile and majority of the transcripts have already been created and localized to the vegetal region in the small stage. In this group, 185 transcripts were observed for *X. laevis* (Figure 4; Supplementary Figure S3; Supplementary Table S2), while 683 for *A. ruthenus* (Figure 5; Supplementary Table S3). The “late vegetal” was characterized by the lack or weak vegetal profile in the early stages compared to the later stages and comprised of two unique subgroups. In the first subgroup, the vegetal transcripts showed relocalization during oogenesis from ubiquitous distribution in very small/small oocytes to the vegetal region in the medium and big stages (56 *X. laevis* and 1136 *A. ruthenus*) (Figures 4, 5; Supplementary Table S2, S3). In the second subgroup, the vegetal transcripts (486 *X. laevis* and 740 *A. ruthenus*) had already formed a slight vegetal gradient (referred to as predefined) in the early stage, but it became more pronounced during the later stages of oogenesis (Figures 4, 5; Supplementary Figure S4; Supplementary Tables S2, S3). We compared these stage dependent vegetal transcripts with those previously described as early, intermediate, and late in the literature. Out of the published maternal transcripts for *X. laevis*, we observed 16 out of 20 correlated as early, 3 out of 9 for intermediate and 8 out of 19 for late (Supplementary Table S4).

#### 3.1.2 Animally localized transcripts

Similar changes in profiles were also observed for the animally localized transcripts. An “early animal” group was detected only for the *A. ruthenus* (722 transcripts), where the profile was already markedly established in the early stage (Figure 5; Supplementary Figure S4; Supplementary Table S3). A “late animal” group was observed in both the *X. laevis* and *A. ruthenus* (Figures 4, 5; Supplementary Figure S3; Supplementary Tables S2, S3). This group contained several subgroups. In the first subgroup, the relocalization from ubiquitous to animal hemisphere during oogenesis was observed between the very small/small and the big oocytes (387 *X. laevis* and 365 *A. ruthenus*). The other subgroup of animal transcripts showed a predefined animal profile in the small oocytes and more pronounced animal profile in the big (168 *X. laevis* and 54 *A. ruthenus*) (Figures 4, 5; Supplementary Tables S2, S3).

#### 3.1.3 Polar localization of transcripts

An interesting group of transcripts was identified in both species and showed an increased localization towards the poles (transcript abundance in both animal and vegetal regions) during oogenesis. In *X. laevis*, 100 different transcripts were enriched at the poles in the small, medium and big oocytes (Figure 4; Supplementary Table S2). In contrast, *A. ruthenus* showed two polar transcript groups (Figure 5; Supplementary Table S3). The first group contained transcripts (120) localized to both poles (animal and vegetal) in the small oocytes and relocalization to vegetal gradient in big. The second group contained transcripts (24) localized preferentially in the animal region of the small oocytes and a polar profile in the big oocytes.

### 3.2 Transcript number alteration during oogenesis responsible for gradient formations

**FIGURE 6:**
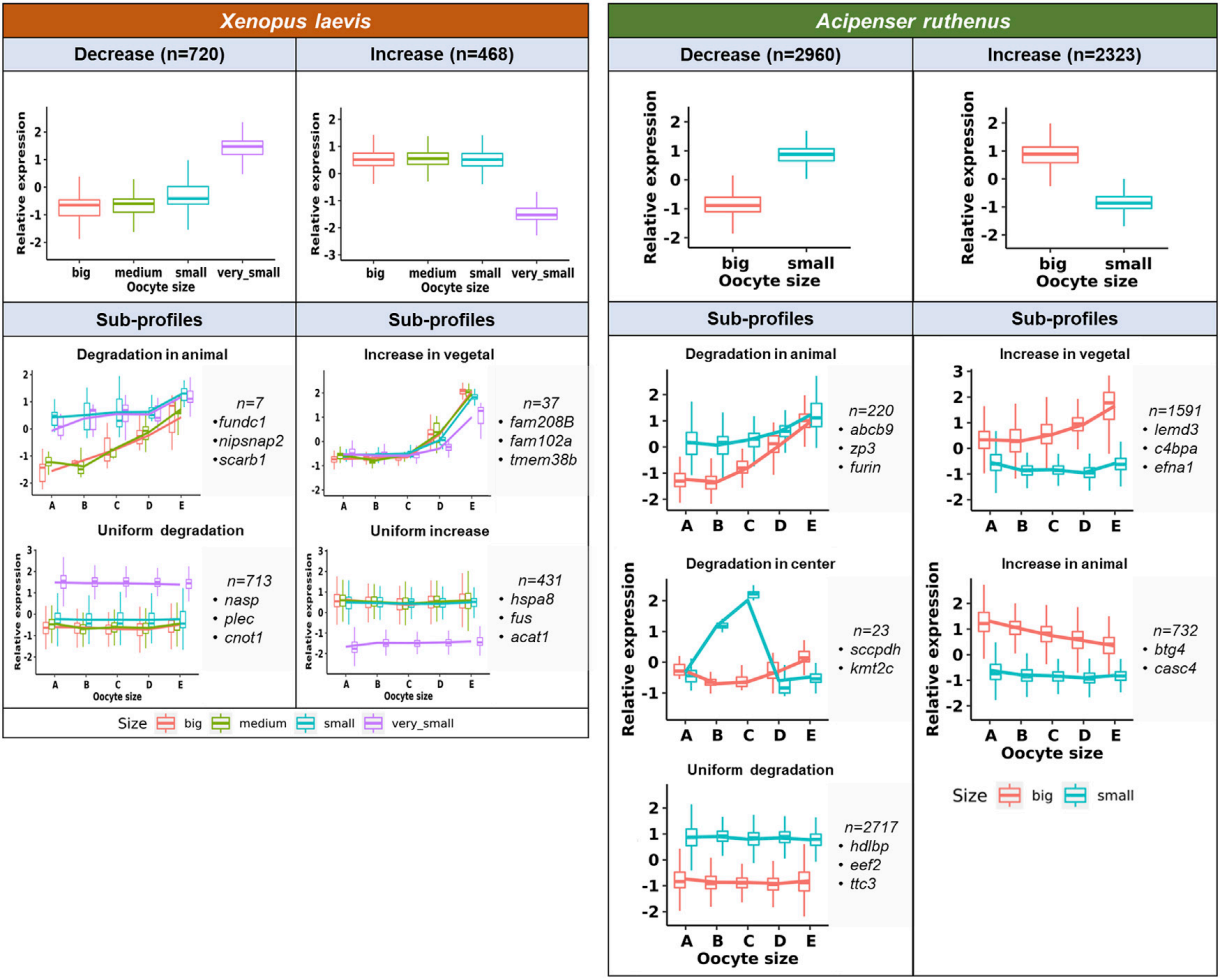
Unique profiles representing the significant total transcript count alterations between the different oocyte sizes of the *Xenopus laevis and Acipenser ruthenus.* The sections correspond to the regions of the oocyte that were cryosectioned, whereby sections A - extremely animal, B - animal, C - central, D - vegetal, E - extremely vegetal.

Transcript count numbers obtained by TOMO-Seq were used to estimate the level of DLTs in particular oocyte stages and to identify significantly degraded (decreased) or *de novo* synthesized (increased) transcripts (Figure 6). There were 9533 and 13024 DLTs (padj <0.1, >20 transcripts in at least one stage) that showed total transcript alterations between stages in the *X. laevis* and *A. ruthenus* respectively. Out of these DLTs, in the *X. laevis* 13% (1188) and in the *A. ruthenus* 41% (5283) showed greater than 1.5x fold alterations (Figure 6; Supplementary Tables S2, S3). These transcripts represented 7% for *X. laevis* and 22% for *A. ruthenus* of the total maternal transcripts (>20 transcripts/oocyte).

It was found that 720 *X. laevis* and 2960 *A. ruthenus* transcripts showed degradation during oogenesis (Figure 6; Supplementary Tables S2, S3). In contrast, the transcript levels of 468 *X. laevis* and 2323 *A. ruthenus* showed an increase during oogenesis (Figure 6; Supplementary Tables S2, S3). In *X. laevis*, the largest differences were observed between the very small and small oocytes and nicely correlated with the PCA results (Figures 3, 6).

Subgroups can be distinguished based on localization profile changes. The first subgroup (7 *X. laevis* and 220 *A. ruthenus*) showed animal degradation leading to vegetal gradients in big oocytes (Figure 6; Supplementary Tables S2, S3). The second subgroup showed an increase (*de novo*) of transcripts within the vegetal region in the big oocytes (37 *X. laevis* and 1591 *A. ruthenus*), resulting in a more profound vegetal profile (Figure 6; Supplementary Tables S2, S3). There were also transcripts that were uniformly degraded without gradient changes (713 *X. laevis* and 2717 *A. ruthenus*) or uniformly synthesized without gradient changes (431 *X. laevis* and 0 *A. ruthenus*) (Figure 6; Supplementary Tables S2, S3). *A. ruthenus* oocytes also showed animal localization in the big oocytes caused by *de novo* synthesis in 732 cases and another subgroup with degradation in the small oocyte center region (23) (Figure 6; Supplementary Tables S2, S3).

### 3.3 Interspecies similarities

**FIGURE 7:**
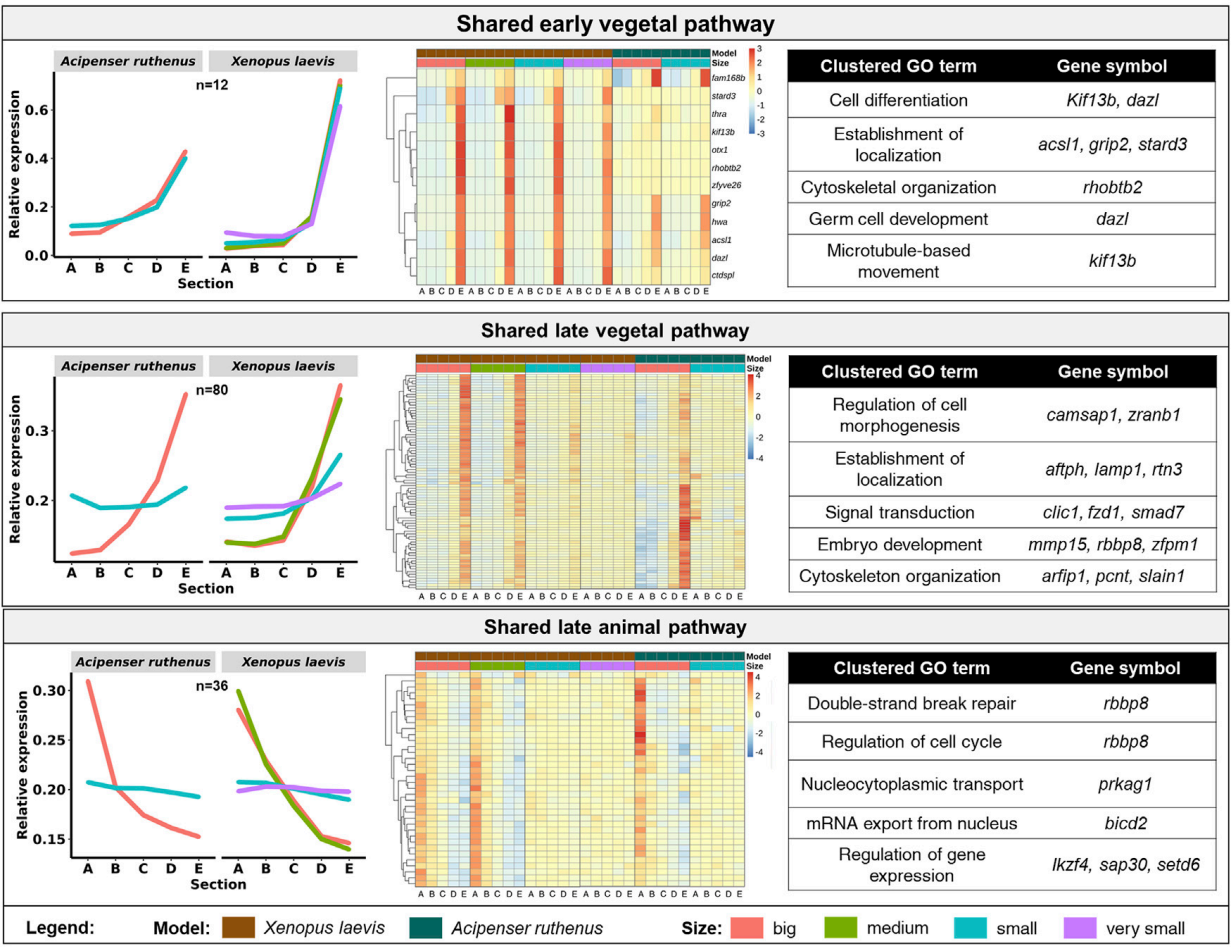
Vegetal and animal transcripts with similar temporal sub-cellular profiles in *Xenopus laevis* and *Acipenser ruthenus*. Heatmap is based on the Z-score of the transcript expression relative to the oocyte stage of the model. The Gene Ontology terms are those found associated with the given group of transcripts. Transcripts were filtered to include only those with either limited (early vegetal < ∼1.5x) or enhanced (late pathways > ∼1.2x) fold differences between stage sections of interest. The sections correspond to the regions of the oocyte that were cryosectioned, whereby sections A - extremely animal, B - animal, C - central, D - vegetal, E - extremely vegetal.

**FIGURE 8:**
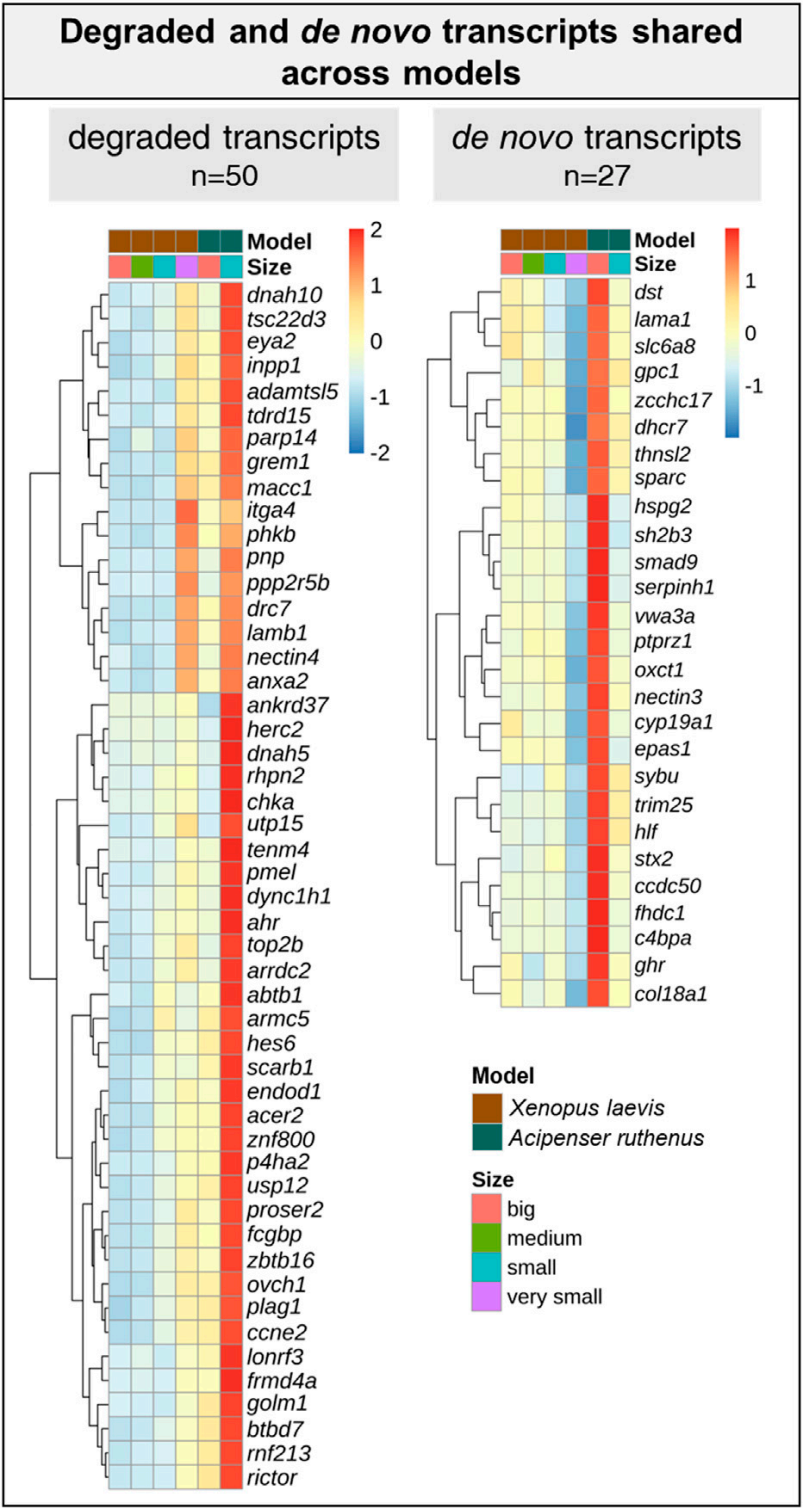
List of transcripts with a reduced or over abundance during the oocyte development between the *Xenopus laevis* and *Acipenser ruthenus*. Heatmap is based on the Z-score of the percentage mean transcript expression relative to each model. Representative transcripts show a minimum of ∼1.5x change relative to a given oocyte stage.

We were able to identify 128 DLTs, that showed similar profiles during oogenesis between *X. laevis* and *A. ruthenus*. Twelve of them showed a stable and steep vegetal gradient already at the very small/small stages (early vegetal), which was preserved to the big oocytes (eg. *dazl* and *grip2* transcripts) (Figure 7; Supplementary Table S5). Other groups of 80 and 36 DLTs showed conserved formation of late vegetal or animal profiles during oogenesis, respectively (Figure 7; Supplementary Tables S6, S7). Gene Ontology terms associated with these conserved DLTs, although insignificant, supported their function in gonad formation and regulation of development in the vegetal region, in contrast to the more nucleus related functions found for the transcripts conserved within the animal region (Figure 7). Levels of transcript counts among the oocyte stages were used to compare conserved degradation or *de novo* transcription during oogenesis (Figure 8; Supplementary Tables S8, S9). The transcripts of 27 genes were found to be newly produced with the majority being observed during the small (*X. laevis*) or big (*A. ruthenus*) stages (Figure 8; Supplementary Table S9). On the other hand, transcripts of 50 genes were degraded during oogenesis (Figure 8; Supplementary Table S8).

## 4 Discussion

Oocytes contain a unique set of transcripts coding genes that are important for their growth, meiotic recombination and division, metabolism of nutrients, and directing of early embryonic development (Song et al., 2006; Lyman-Gingerich and Pelegri, 2007). Here, we compared oocytes of representatives from the class *Amphibia* (*X. laevis*) and class *Actinopterygii* (*A. ruthenus*). It is known that both species show similarities during their oocyte development (Raikova, 1973; Raikova, 1974) and therefore there was an expectation that this may also be reflected in a high conservation of their maternal transcript localization. Additionally, microscopically it can be seen that the two models share similar transition of their nuclear region from a central location to a more animal localization from the transition from small (early vitellogenic) oocyte to the big (advanced vitellogenic) oocyte (Dumont, 1972).

Most of our knowledge about transcript localization have focused mainly on the vegetal localization, and primarily within the *X. laevis* model. This has led to the elucidation of three main pathways, those being the METRO, intermediate and Late pathways (Forristall et al., 1995; Kloc and Etkin, 1995). However, there is still controversy about the temporal regulation of each pathway, and it is common that pathways may actually overlap during multiple stages of the oocyte development. In addition, there is a lack of knowledge about animal localization and the mechanism driving it. *In situ* hybridization has been a prime method for studies of transcript localization during oogenesis and a few transcripts have already been analyzed (Kloc and Etkin, 1995, 1998; reviewed by King et al., 1999; Schroeder and Yost, 1996). However, large-scale analyses offer the potential to assess the full transcriptomic landscape and therefore offer a clearer picture on the complex drivers for development. Using TOMO-Seq approach, we measured the spatial transcript localization of nearly 16000 and 22000 transcripts in *X. laevis* and *A. ruthenus*, respectively. Early oocytes (very small in *X. laevis* and small in *A. ruthenus*) showed 4-times lower polarity (less localized DLTs) compared to larger oocytes. *A. ruthenus* oocytes showed ∼10-times more DLTs compared to *X. laevis*, which suggest that it is much more polarized and that gradients are steeper. We observed the same result while analyzing mature eggs for these species (Naraine et al., 2022). This difference in the amount of DLTs may also be a reflection in the number of transcripts and also size differences between the two models, whereby the *A. ruthenus* has about 10000 more transcripts and also is ∼1.6x bigger than the *X. laevis*.

In this study, we did not analyze early oocytes (I-II stages), because they lack pigmentation allowing oocyte polarity determination (Dumont, 1972). Transcripts utilizing METRO pathway are already localized to the vegetal pole during these early stages and remain vegetally localized until oocyte maturation. Known PGC markers such as *dazl* and *grip2* are among conserved extremely vegetal group localized early in both species suggesting conserved function during development. Transcripts (eg. *dnd1*, *plin2*, *rbpms*), which are supposed to utilize intermediate pathway, are localized in the Balbiani body at the early stages of oogenesis and continue their migration using the Late pathway during stages II-V (Chan et al., 1999; reviewed by Houston, 2013). These could be potentially transcripts labelled as predefined in Figures 4, 5. Late pathway shifts RNAs towards the vegetal pole from stage III oocytes (Forristall, et al., 1995; Kloc and Etkin, 1998; reviewed by King et al., 1999) and we can speculate that late vegetal localization are transcripts homogenous during early and becoming vegetal in the late stages of oogenesis. We expect an overlap of intermediate pathway with METRO pathway based on the selected oocyte stages. Similar changes of localization profiles were found also in *A. ruthenus.* Both early and late vegetal localization appears for hundreds of DLTs.

Little is known about animal localization. In our previous study we hypothesized that animal DLTs are produced in the oocyte germinal vesicle, which is located in the animal hemisphere, and DLTs diffuse during oogenesis, without active transport, producing the observed peak in the first 1/3 of the egg from the animal pole (Sindelka et al., 2010, 2018). Previously we had identified an additional animal localization profile called extremely animal, which formed a gradient from animal to vegetal poles and we speculated that extremely animal DLTs are localized actively using an unknown mechanism. Using DESeq2 normalization (median-of-ratios method) we removed the majority of the animal transcripts (forming >90% of total maternal DLTs in mature eggs) and focused primarily on extremely localized transcripts. We found that both species utilized the animal pathway. The majority of animal localization in *X. laevis* appeared during the late stages of oogenesis, while in *A. ruthenus* we revealed two potential pathways: early and late. Animal localization during the late phases of *X. laevis* oogenesis has been already suggested by other studies (Yisraeli et al., 1990; Schroeder and Yost, 1996; reviewed by Schnapp at el., 1997). In *A. ruthenus*, the early group is localized into the animal pole already in the small oocytes and their animal gradient become steeper during oogenesis. The late group is localized ubiquitously in the small oocytes and the animal gradient is formed during oogenesis. We identified a potentially interesting group of DLTs, which were localized to both poles of the oocytes: animal and vegetal at the same time. To reveal whether there is biological relevance of polar localization or if it is just a result of data normalization would require more thorough analysis using alternative approaches such as *in situ* hybridization and functional validation.

Changes in the transcript levels during oogenesis indicate a dramatic dependence of gene expression during oocyte maturation and tight regulation of oocyte polarity. The largest difference was observed comparing very small (*X. laevis*) and small (*A. ruthenus*) oocyte with the other stage, while only minor differences can be observed when comparing small and medium sizes of *X. laevis* oocytes. Transcripts coding hundreds (*X. laevis*) or thousands (*A. ruthenus*) of maternal genes were degraded or *de novo* synthesized during oogenesis and in many cases led to changes in localization profiles. Vegetal gradients were formed preferentially in *X. laevis* in contrast to both animal and vegetal gradients formation caused by degradation or *de novo* transcription in *A. ruthenus*. Such extensive transcriptional activity and *de novo* transcription was also described by Meneau et al. (2020) and Session et al. (2016) for the *X. laevis*, whereby they observed that between stages I-II and stages V-VI, 1557 transcripts showed an increased abundance in the oocytes, while 17 transcripts showed a decreased abundance. This correlates with what we observed whereby *de novo* and degradation of transcripts were observed from stages IV (small) to late stage V (big), relative to the stage III (very small).

The overall overlap between the two models in temporal localization profiles was relatively small. However, these few conserved DLTs are known to be important for early embryonic development. Group of vegetal DLTs, which are formed early (*dazl* and *grip2*), but also many DLTs localized late in oogenesis (*dnd1*) are known determinants of PGC formation or early development regulators. In contrast, conserved animally localized DLTs are important for cell cycle regulation and other nuclear processes.

In summary our data reflect highly dynamic changes at the transcript level during oogenesis. Precise spatial and temporal regulation of biomolecule localization are required for final oocyte quality, which is potentially crucial for normal development of the fast-dividing embryos, such as the studied examples of frogs and fishes. We found in our results examples for most of the theoretical scenarios including relocalization in space and time and spatially controlled degradation of transcripts. We believe that our data will serve as an essential initial source for target identification in oogenesis and future consequential studies will benefit from using alternative approaches to analyze gene functions during oogenesis and early embryogenesis.

## Data Availability

All raw and processed sequencing data generated in this study have been submitted to the National Center for Biotechnology Information’s Gene Expression Omnibus (GEO) database, at https://www.ncbi.nlm.nih.gov/geo/, and can be accessed with the GEO deposition number: GSE211415 (Xenopus laevis) and GSE211412 (Acipenser ruthenus).
