# Supplementary material for: Comparison of RNA localization during oogenesis within Acipenser ruthenus and Xenopus laevis
Source: Front Cell Dev Biol. 2022 Sep 20;10:982732. doi: 10.3389/fcell.2022.982732 (PMC9531136; doi:10.3389/fcell.2022.982732)
Supplement: Supplementary file 2 [file Image1.pdf]

This document includes Supplemental Figures S1-S4.

Supplemental Figures

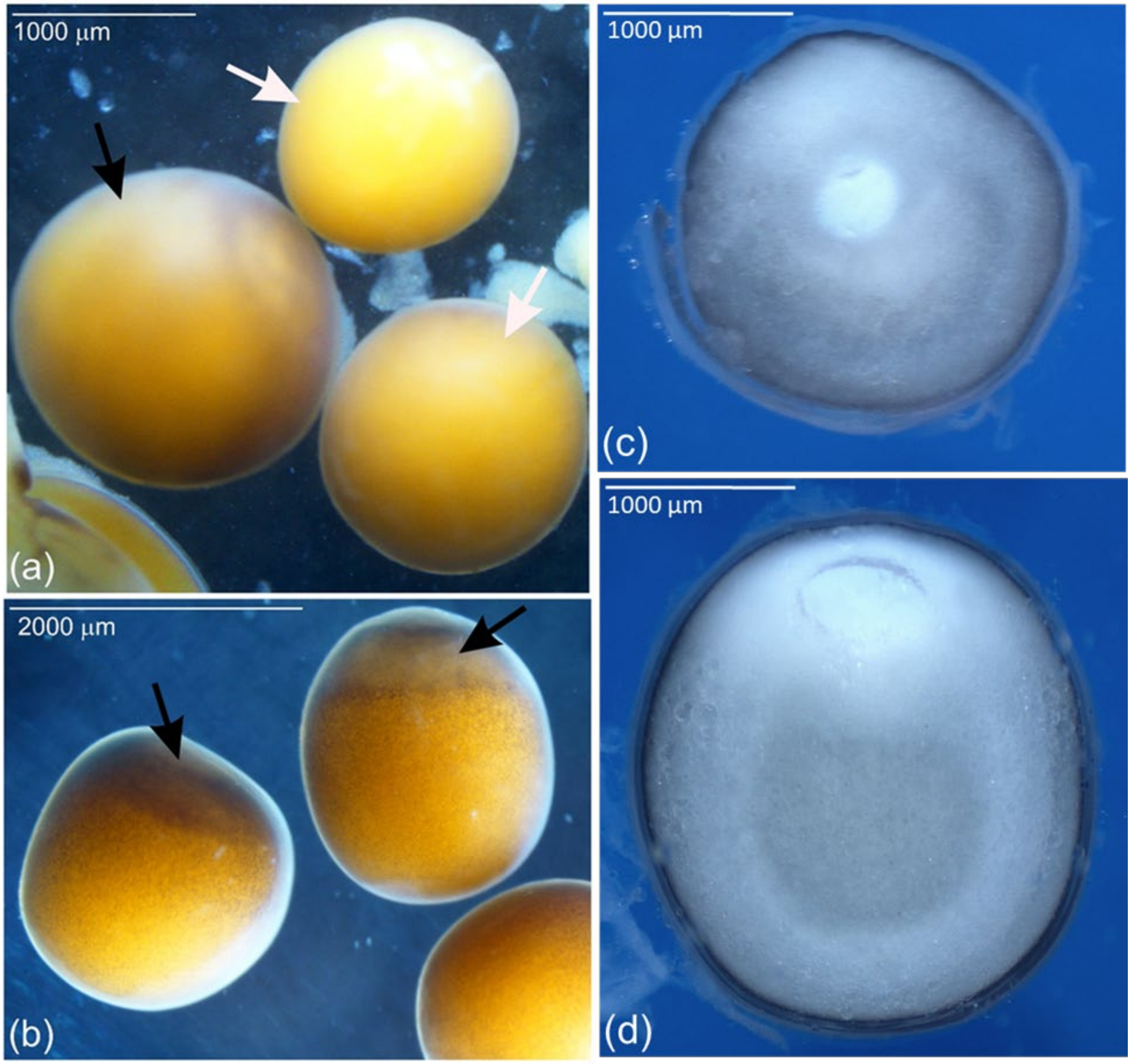

Figure S1. **Ovarian follicles containing early (small) and advanced (big) vitellogenic oocytes of *Acipenser ruthenus*.** The stereo microscope images show, a) white arrow – follicles containing small oocytes, black arrow – follicle containing big oocyte, b) black arrow – animal hemisphere of the big oocytes, c) sectioned small oocyte along the animal-vegetal axis, d) sectioned big oocyte along the animal-vegetal axis.

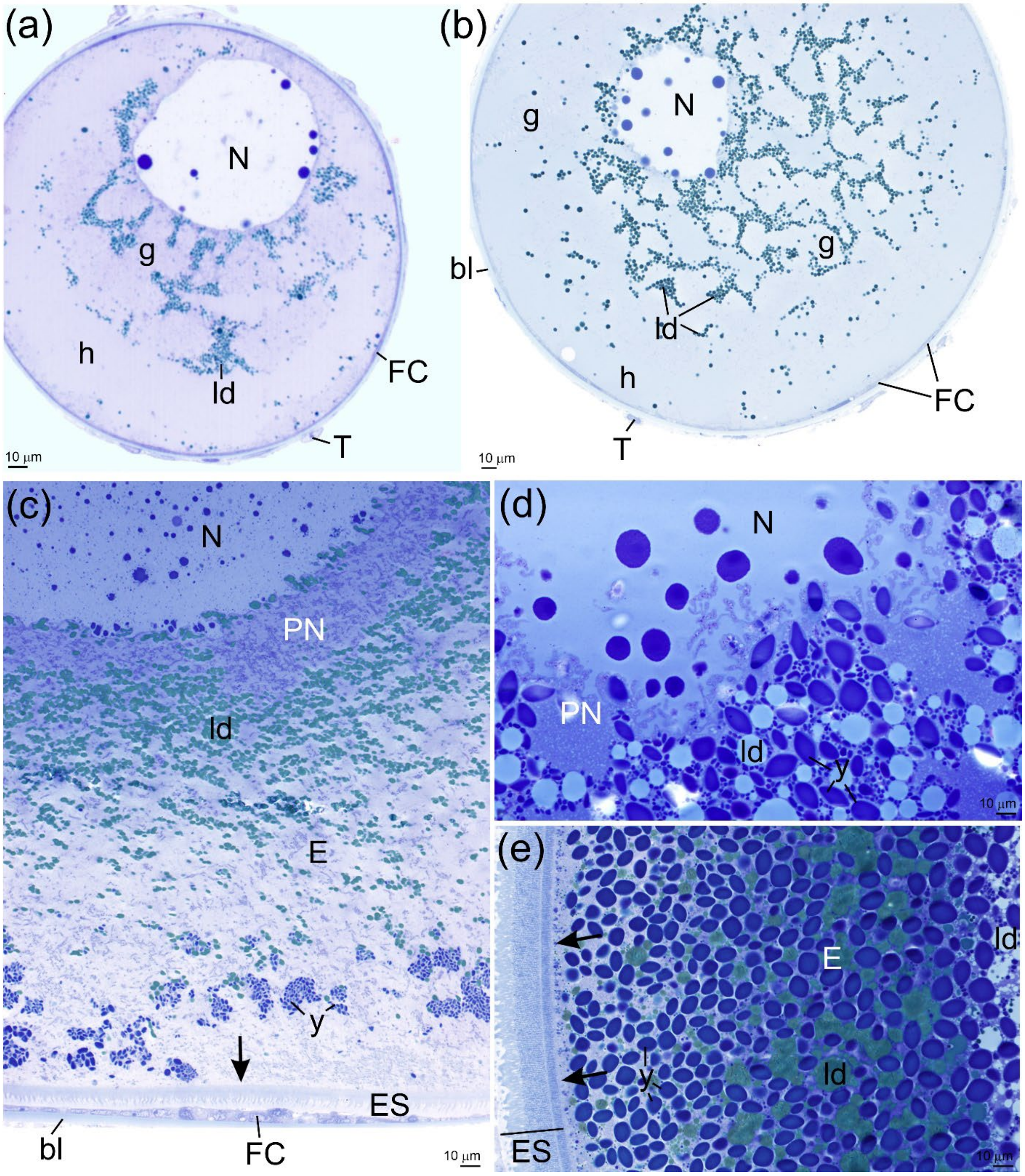

Figure S2. **Microscopic characterization of the oocytes of *Acipenser ruthenus*. a)** Early previtellogenic ovarian follicle. N – oocyte nucleus, g – granular ooplasm, h – homogeneous ooplasm, ld – lipid droplets in the lipid body, FC – follicular cell, bl – basal lamina, T – thecal cell; **b)** Midprevitellogenic ovarian follicle. N – oocyte nucleus, g – granular ooplasm, h – homogeneous ooplasm, ld – lipid droplets in the lipid body, FC – follicular cells, bl – basal lamina, T – thecal cell; **c)** Early vitellogenic ovarian follicle (small oocyte). N – oocyte nucleus in the oocyte center, PN – perinuclear ooplasm, E – endoplasm, arrow – periplasm, ld – lipid droplets, y – yolk platelets,

ES – eggshell, FC – follicular cell, bl – basal lamina, T – thecal cell. Light microscope; **d)** Central region of the oocyte during advanced vitellogenesis (big oocyte). N – oocyte nucleus, PN – perinuclear ooplasm, ld – lipid droplet, y – yolk platelets; **e)** Fragment of the ovarian follicle during advanced vitellogenesis (big oocyte). E – endoplasm, arrows – periplasm, ES – eggshell, ld – lipid droplets, y – yolk platelets.

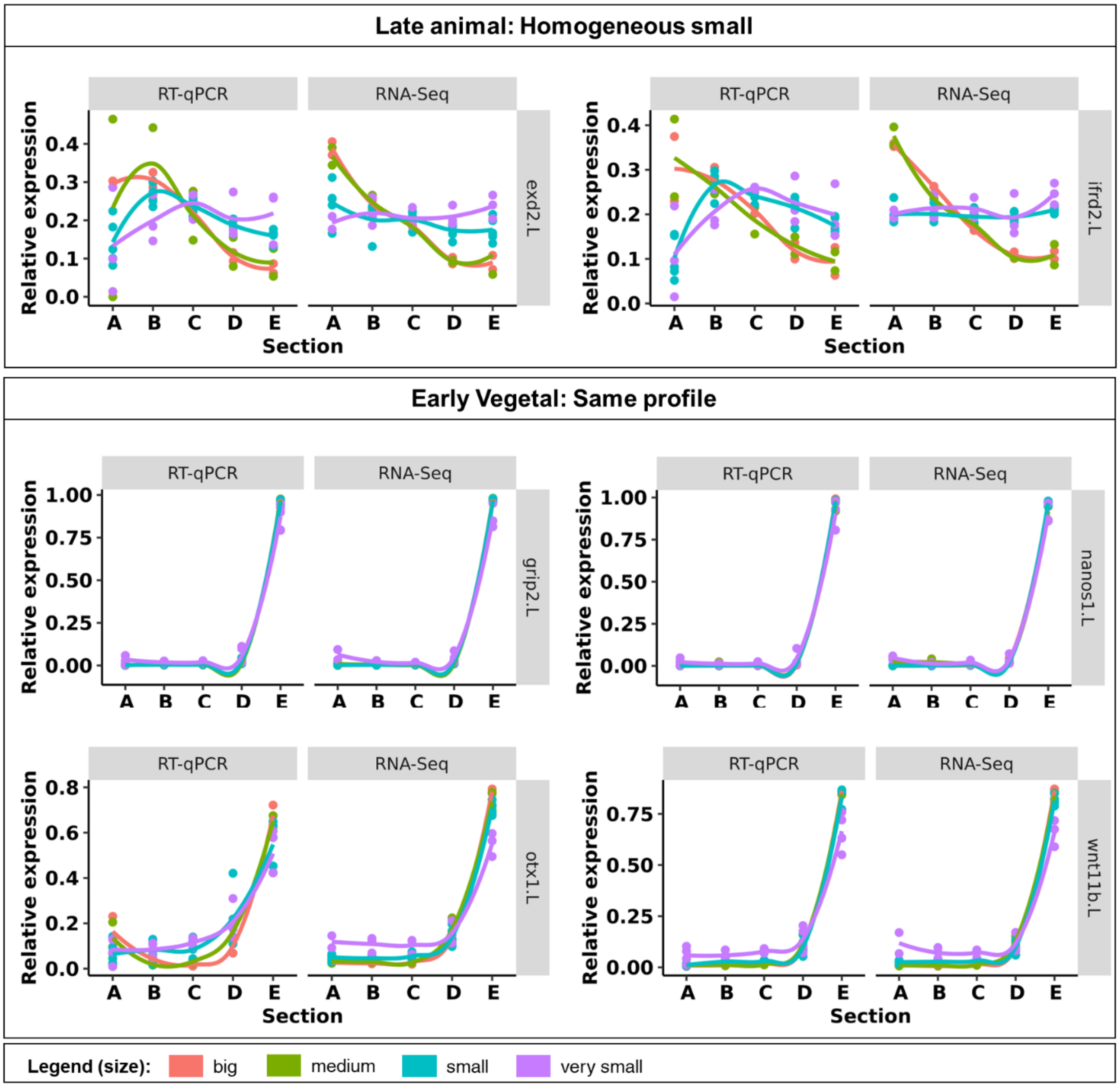

Figure S3. **Comparison of asymmetrical distribution of *Xenopus laevis* maternal transcripts across the different oocyte stages using RT-qPCR and RNA-Seq.** The sections correspond to the regions of the oocyte that were cryosectioned, whereby sections A - extremely animal, B - animal, C - central, D - vegetal, E - extremely vegetal.

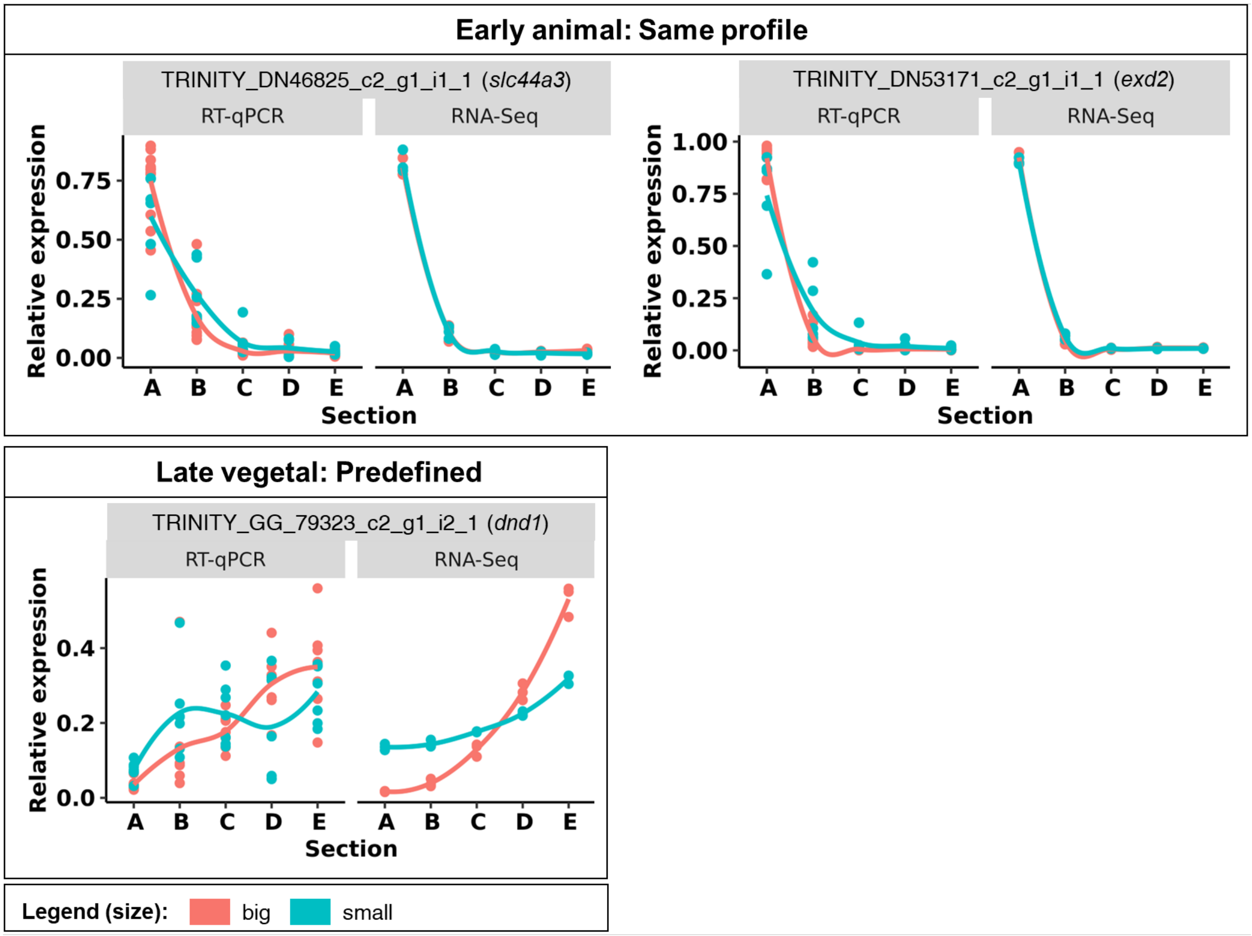

Figure S4. **Comparison of asymmetrical distribution of *Acipenser ruthenus* maternal transcripts across the different oocyte stages using RT-qPCR and RNA-Seq.** The sections correspond to the regions of the oocyte that were cryosectioned, whereby sections A - extremely animal, B - animal, C - central, D - vegetal, E - extremely vegetal.
